# Photo-functionalized TiO_2_ film for facile immobilization of EpCAM antibodies and efficient enrichment of circulating tumor cells

**DOI:** 10.3389/fphar.2023.1126602

**Published:** 2023-02-28

**Authors:** Huan Deng, Xiangqin Liu, Jie Chen, Yi He, Lanke Lin, Xin Liu, Jiang Chen, Xiaoqi Liu

**Affiliations:** ^1^ College of Medical Technology, Chengdu University of Traditional Chinese Medicine, Chengdu, China; ^2^ Department of Laboratory Medicine and Sichuan Provincial Key Laboratory for Human Disease Gene Study, Sichuan Provincial People’s Hospital, University of Electronic Science and Technology of China, Chengdu, China; ^3^ Department of Core laboratory, Sichuan Provincial People’s Hospital, University of Electronic Science and Technology of China, Chengdu, China; ^4^ Department of Blood Transfusion, Sichuan Academy of Medical Sciences & Sichuan Provincial People’s Hospital, Chengdu, China; ^5^ The Department of Ophthalmology, Sichuan Provincial People’s Hospital, University of Electronic Science and Technology of China, Chengdu, China; ^6^ Sichuan Provincial Key Laboratory for Human Disease Gene Study, Center for Medical Genetics, Sichuan Academy of Medical Sciences & Sichuan Provincial People’s Hospital, University of Electronic Science and Technology of China, Chengdu, China; ^7^ Research Unit for Blindness Prevention of Chinese Academy of Medical Sciences (2019RU026), Sichuan Academy of Medical Sciences & Sichuan Provincial People’s Hospital, Chengdu, Sichuan, China

**Keywords:** photo-functionalized TiO_2_, EpCAM antibodies, circulating tumor cells (CTCs), capture, tumor screening

## Abstract

The highly efficient capture of circulating tumor cells (CTCs) in the blood is essential for the screening, treatment, and assessment of the risk of metastasis or recurrence of cancer. Immobilizing specific antibodies, such as EpCAM antibodies, on the material’s surface is currently the primary method for efficiently capturing CTCs. However, the strategies for immobilizing antibodies usually have the disadvantages of requiring multiple chemical reagents and a complex pre-treatment process. Herein we developed a simple strategy for the immobilization of EpCAM antibodies without additional chemical reagents. By utilizing the positive charge property of the photo-functionalized titanium dioxide (TiO_2_), the negatively charged carboxyl terminal of EpCAM antibodies was immobilized by electrostatic interaction, allowing the antibodies to expose the antigen binding site fully. The experimental results showed that the photo-functionalized TiO_2_ surface had a marked positive charge and super-hydrophilic properties that could immobilize large amounts of EpCAM antibodies and keep excellent activity. CTCs capture experiments *in vitro* showed that the EpCAM antibodies-modified photo-functionalized TiO_2_ could efficiently capture CTCs. The results of blood circulation experiments in rabbits showed that the EpCAM antibodies-modified photo-functionalized TiO_2_ could accurately capture CTCs from the whole body’s blood. It was foreseen that the strategy of simple immobilization of EpCAM antibodies based on photo-functionalized TiO_2_ is expected to serve in the efficient capture of CTCs in the future.

## 1 Introduction

Cancer is a significant public health threat, inducing more than 10 million deaths in 2020 worldwide (Sung et al., 2021). Circulating tumor cells (CTCs) are cancer cells that are shed from the primary lesion of a solid tumor, enter the circulation and/or lymphatic system, and translocate to distant tissues to form a secondary tumor (Plaks et al., 2013; Castro-Giner and Aceto, 2020). Increased CTCs in the blood are associated with tumor metastasis and the short interval between tumor recurrences (Chaffer and Weinberg, 2011). Therefore, as a new target for “liquid biopsy,” CTCs are of great clinical significance in assessing patients for postoperative monitoring, postoperative adjuvant therapy, and guiding the development of targeted treatment plans (Lin et al., 2018).

Currently, the enrichment methods for CTCs can be divided into *in vitro* and *in vivo* enrichment methods. The common *in vitro* CTCs enrichment methods include microfluidic devices (Su et al., 2019; Shi et al., 2021; Abdulla et al., 2022; Liu et al., 2022), photoelectrochemical platforms (Parker et al., 2018; Xu et al., 2021), immunomagnetic beads (Mohamadi et al., 2015; Wang et al., 2019; Li et al., 2020; Liang et al., 2020; Zhou et al., 2022), and new patterns (Jahangiri et al., 2020; Kang et al., 2021; Zhang et al., 2021; Wang et al., 2022b; Wang et al., 2022c) et al. False negative test results are the main problem that plagues these *in vitro* methods, because CTCs are very scarce in peripheral blood. There are only a few CTCs in 1 mL of blood, but there are millions of blood cells (Paterlini-Brechot and Benali, 2007). Therefore, materials that can efficiently enrich CTCs from a few mL of collected blood are crucial to improving the sensitivity and accuracy of CTCs detection. Compared to the *in vitro* methods, the *in vivo* enrichment methods, such as vein indwelling needles (Zhang et al., 2015), flexible electronic catheters (Wang et al., 2022a), plasmon resonance fiber probes (Zhu et al., 2022), and black phosphorus-modified intravenous catheters (Wang et al., 2020), are expected to enrich more CTCs from whole body blood, thus reducing the number of false negative results. However, these methods need to implant the CTC-enrich materials into the body, which may lead to various host reactions such as coagulation and inflammation. Therefore, beyond the ability to efficiently enrich CTCs, the *in vivo* enrichment method places additional requirements on the materials, namely, excellent biocompatibility.

Furthermore, a common problem faced by both *in vivo* and *in vitro* enrichment methods is that the strategy for the immobilization of CTC-capture antibodies on the surface of the material needs to be further simplified. For example, in the immobilization of EpCAM antibodies (the most used CTCs capture antibody), Parker et al. (2018) used oligo oxide molecules to attach antibodies on the silicon surface *via* an N, N-disuccinimidyl carbonate activating group. Wang et al. (2020) used the carbodiimide crosslinking agent 1-(3-dimethylaminopropyl)-3-ethyl carbodiimide hydrochloride to conjugate EpCAM antibodies *via* amido linkage on the surface of the intravenous catheter. Stott et al*.* (2010) used coupling agents attached to avidin and then coupled them to biotinylated EpCAM antibodies to functionalize the microfluidic device. All of these methods required using complex tools and toxic chemical reagents. Therefore, it is of great significance to develop a strategy to fix EpCAM antibodies more simply.

Titanium dioxide (TiO_2_) is a ceramic material popular in orthopedics and blood contact materials for its excellent biocompatibility. Therefore, TiO_2_ meets the stringent bio-safety and biocompatibility required by CTCs *in vivo* capture materials. Chen et al*.* have shown that TiO_2_ irradiated by ultraviolet light (UV) has better anticoagulation compared with unirradiated TiO_2_ (Chen et al., 2014; Chen et al., 2015). When TiO_2_ was irradiated by UV, the electrons in the valence band transitioned, and the positive holes were generated at simultaneously, forming the electron-hole pairs (Wang et al., 1997; Zubkov et al., 2005; Hori et al., 2010a). Among them, photogenerated electrons have a strong oxidation capacity, while photogenerated holes have a reducing capacity (Hori et al., 2010a; Pelaez et al., 2012), resulting in the transformation of TiO_2_ from biologically inert to biologically active and causing a self-cleaning effect (Hori et al., 2010b; Ueno et al., 2010), which desorbs many inert hydrocarbons adsorbed on the surface, exposing a more positive charged surface of TiO_2_, and increasing the hydrophilicity of the surface (that is, increasing the surface energy) (Takeuchi et al., 2005; Zhang et al., 2008; Hori et al., 2010a; Iwasa et al., 2010). Therefore, the Photo-functionalized TiO_2_ could improve the adsorption of the proteins through electrostatic adsorption and thermodynamics.

**FIGURE 1:**
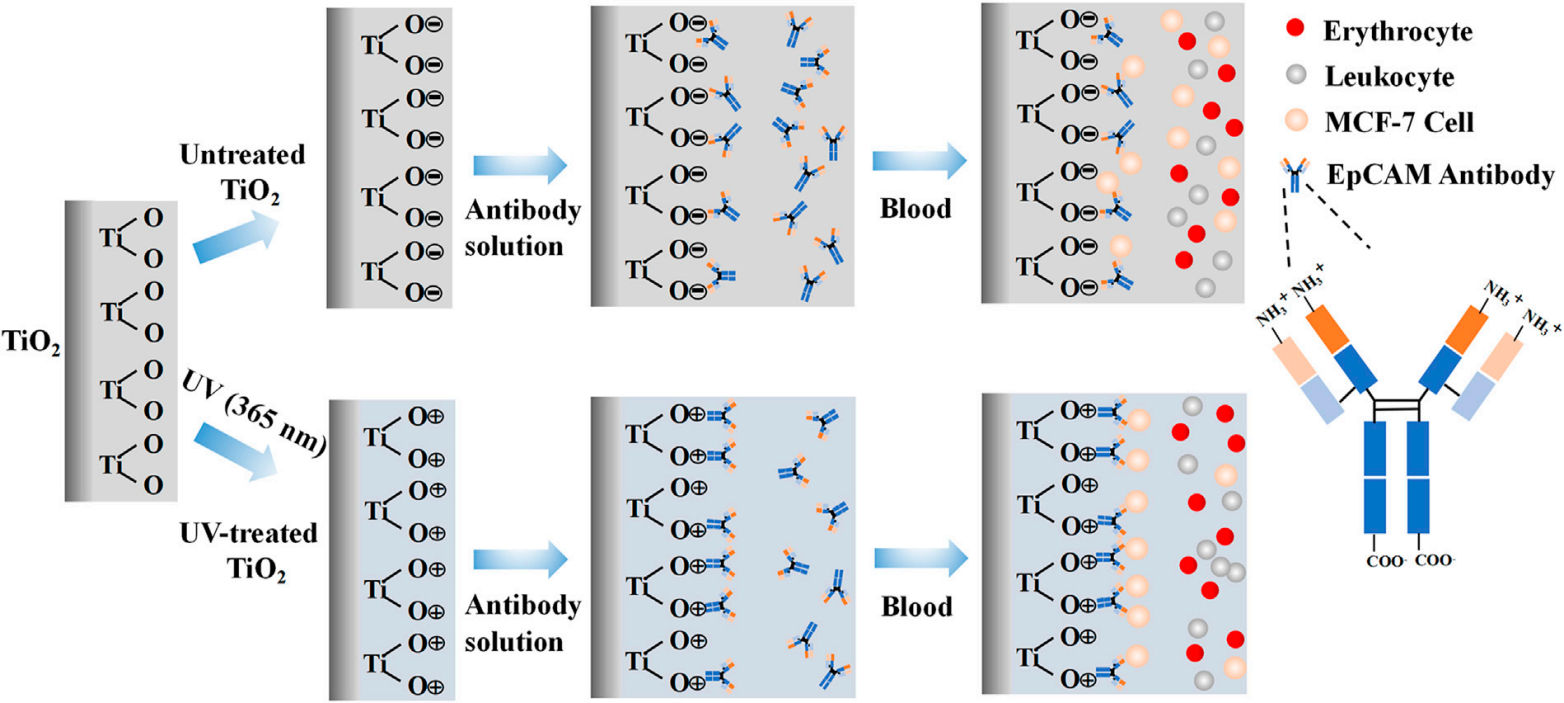
Hypothetical mechanism of electrostatic interaction between EpCAM antibodies and photo-functionalized TiO_2_ surface, and schematic diagram of cells capture.

EpCAM antibodies are composed of multiple amino acids. The negatively charged carboxyl terminal is the constant region of the antibody, and the positively charged amino terminal is the variable region of the antibody, which is also the binding site for the CTCs surface antigen. At physiological pH (7.0), the surfaces of TiO_2_ are known to be negatively charged (Ellingsen, 1991; Hori et al., 2010a). Therefore, we hypothesized that the photo-functionalized TiO_2_ surface could bind to the negatively charged carboxyl terminal of the antibody through an electrostatic mechanism to immobilize the EpCAM antibodies and expose the binding site of the EpCAM antibodies to the antigens, thereby achieving highly sensitive capture CTCs (Figure 1). Compared with the traditional method of chemically grafting antibodies, photo-functionalized TiO_2_ as a substrate to bind EpCAM antibodies has the characteristics of simplicity and no need to use toxic chemical reagents. As mentioned above, TiO_2_ has excellent biocompatibility. In addition, TiO_2_ can also be used on the surface of various inorganic materials by the physical vapor deposition method. Therefore, the method may be suitable for constructing various devices for capturing CTCs *in vitro* (such as magnetic beads and silicon-based photoelectrochemical platforms) and *in vivo* (stainless steel indwelling needles).

In this study, we characterized the surface physicochemical properties of photo-functionalized TiO_2_ by X-ray photoelectron spectroscopy, water contact angle measurement meter, and potentiometric analysis. Then we immobilized the EpCAM antibodies on the photo-functionalized TiO_2_. After that, we comprehensively studied the CTCs-capture efficiency of the EpCAM antibodies-modified photo-functionalized TiO_2_ *in vitro* and *in vivo* dynamic environments to evaluate the application potential of the strategy in various typical CTCs capture scenes.

## 2 Materials and methods

### 2.1 Materials

Human breast cancer cells (MCF-7) were purchased from Chuan Qiu Biotechnology Company Limited (Shanghai). EpCAM monoclonal antibody was purchased from Proteintech Group, Inc., (Wuhan). Rhodamine stain solution was purchased from Sigma-Aldrich (United States). The CFDA SE Cell Proliferation Assay and Tracking Kit were purchased from Beyotime Biotechnology Company Limited (Shanghai). TiO_2_ nanoparticles were purchased from Sigma-Aldrich (United States).

### 2.2 Construction of CTCs capture materials

Anatase TiO_2_ films were prepared on the Si substrate by an unbalanced magnetron sputtering equipment (UBMS450, Southwest Jiaotong University), according to the deposition parameters of Cui et al. (2021) The TiO_2_ films were cut into 0.7 cm × 0.7 cm pieces and placed in a dark environment for 1 month to stabilize the chemical properties of the surface of the samples. The TiO_2_ films were then irradiated for 1 h at 365 nm UV intensity using a model URE-2000/25-T9 lithography machine (Institute of Optics and Electronics, Chinese Academy of Sciences, China) with a UV intensity of 10 mW/cm^2^. UV-irradiated TiO_2_ films (UV-TiO_2_) and unirradiated TiO_2_ films (UNT-TiO_2_) were separately placed in 24-well plates. The EpCAM antibody solution was diluted with phosphate buffer saline (PBS) to make antibody dilutions at concentrations of 0, 0.001, 0.01, and 0.1 mg/mL, which were prepared and ready to use. The UV-TiO_2_ groups and UNT-TiO_2_ groups were then incubated with different concentrations of antibody solutions for 5 min at room temperature. After the incubation, the samples were washed 3 times with PBS to remove the antibodies that did not adhere firmly. Finally, the samples were stored at 4°C. These prepared samples are respectively represented as UV-TiO_2_-0, UV-TiO_2_-0.001, UV-TiO_2_-0.01, UV-TiO_2_-0.1, and UNT-TiO_2_-0, UNT-TiO_2_-0.001, UNT-TiO_2_-0.01, UNT-TiO_2_-0.1.

### 2.3 Characterization of TiO_2_ film

Atomic Force Microscope (AFM; Nano Navi E-Sweep, Hitachi, Japan) was used to observe the surface morphologies of the samples. X-ray photoelectron spectroscopy (XPS; XSAM800, Kratos Ltd., United Kingdom) was performed to detect the changes in the surface chemical state of the samples before and after antibody adsorption. The hydrophilicity of the samples was detected by a water contact angle measurement meter (WCA; JY-82, Kruss, Germany). A Zeta electric potential analyzer (ZEN3600, Malvern Nano ZS, United Kingdom) was employed to detect the change in the surface charge of the samples (Due to the requirements of the detection equipment, the TiO_2_ films were replaced with TiO_2_ nanoparticles. All other processing factors were the same as above).

### 2.4 *In vitro* capture of CTCs

Each prepared sample was placed in a 24-well plate, and MCF-7 cells were diluted to 10^5^ cells/mL in the DMEM medium. Then 500 µL of cell suspension was added to each sample and placed on a shaker for 40 min. After 40 min, they were washed 3 times with PBS to remove uncaptured cells, followed by 2.5% (v/v) glutaraldehyde for fixation. Finally, cells captured on the surface of different samples were stained with rhodamine stain and observed under a fluorescent microscope (IX51, Olympus, Japan).

### 2.5 Dynamic capture of CTCs *in vitro*

Foldable Ti foils (0.7 cm × 1 cm) covered with TiO_2_ films were used to test the capture efficiency of CTCs in the blood flow state. A chandler loop system (CJ23, Sichuan Academy of Medical Sciences—Sichuan Provincial People’s Hospital) was used to simulate blood flow to capture CTCs. The chandler loop system can better simulate extracorporeal blood circulation and rotate at a certain speed in a temperature-controlled environment to simulate blood flow conditions. The medical catheters containing fresh whole blood (collected in an ethically approved manner from healthy people at the Sichuan Provincial People’s Hospital) with MCF-7 cells (labelled in advance using the CFDA SE fluorescent stain) were connected to the chandler loop system to form a closed circulatory system (Figure 5A). The TiO_2_ foils from the UV-TiO_2_-0, UV-TiO_2_-0.1, and UNT-TiO_2_-0, UNT-TiO_2_-0.1 groups were rolled into separate medical catheters, with each TiO_2_ foil tightly attached to the inner wall of the catheter, and based on the catheter diameter of the chandler loop system and the flow rate of a human arm vein, the liquid flow of the chandler loop system was set to 50 mL/min, the temperature was set to 37°C and cycled for 40 min. Afterward, the samples were gently removed, washed 3 times in PBS, and immediately observed under a fluorescent microscope.

### 2.6 *In vivo* capture of CTCs

All animal experiments were performed in accordance with Chinese regulations on laboratory animal management. New Zealand White rabbits weighing 4.0–4.5 Kg were used. The UV-TiO_2_-0.1 and UNT-TiO_2_-0.1 were selected for the test, and UV-TiO_2_-0 and UNT-TiO_2_-0 were used as controls. The samples were rolled into separate medical catheters, with each sample tightly attached to the inner wall of the catheter. One side of the catheter was connected to the carotid artery of the rabbit and the other to the jugular vein, forming a closed circulatory system (Figure 6A). After successful connection, 1 mL of MCF-7 cells (labelled in advance using the CFDA SE fluorescent stain) were injected from the rabbit’s ear vein. After 40 min of cycling, the samples were gently removed, washed 3 times in PBS, and immediately observed under a fluorescent microscope.

### 2.7 Statistical analysis

One-way ANOVA of SPSS 26.0 software was performed to assess statistical differences between the sample groups. *p < 0.05 indicated significance. Three independent samples were used for each experimental sample group if not otherwise indicated.

## 3 Results and discussion

### 3.1 Characterization of TiO_2_ film

**FIGURE 2:**
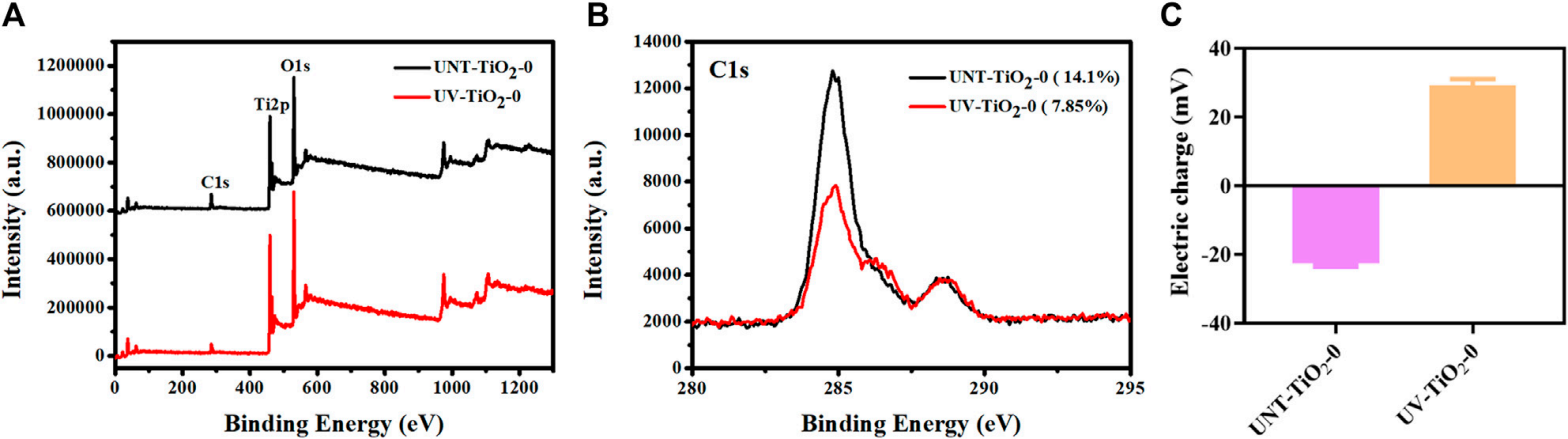
**(A)** XPS full spectrum of elements on TiO_2_ surface before and after UV irradiation. **(B)** XPS high-resolution spectra of C1s on TiO_2_ surfaces before and after UV irradiation. **(C)** Charge changes on the TiO_2_ surface before and after UV irradiation.

Figures 2A, B showed the XPS spectra of C1s of the TiO_2_ surface before and after the UV irradiation. The content of the carbon (C) element on the unirradiated TiO_2_ surface was 14.1%, while after UV irradiation, the content of the C element decreased to 7.85%. This might be due to the self-clean effect, which decomposed the hydrocarbons adsorbed on the TiO_2_ surface (Takeuchi et al., 2005; Zhang et al., 2008). The decrease of the C element indicated the exposure of the clean TiO_2_ surface, which might bind more of the antibodies.

As shown in Figure 2C, the changes in charge of the TiO_2_ surface before and after UV irradiation were examined. The TiO_2_ surface was negatively charged before UV irradiation, while the surface showed a positive charge after UV irradiation. This positively charged surface could facilitate the carboxyl terminal of the antibody to conjugate with the TiO_2_ surface through electrostatic interaction, thus fully exposing the antibody’s antigen-binding site. However, there was also a problem that there are usually many amino and carboxyl groups in the side chain of an antibody, which would affect the adhesion mode of antibodies and TiO_2_. That is, in addition to the binding method shown in Figure 1, antibodies might also be combined with TiO_2_ through carboxyl groups on the side chain, in the form of lying on the side, which would affect the exposure of the antigen-binding site of the antibody and hence the effectiveness of cell capture.

**FIGURE 3:**
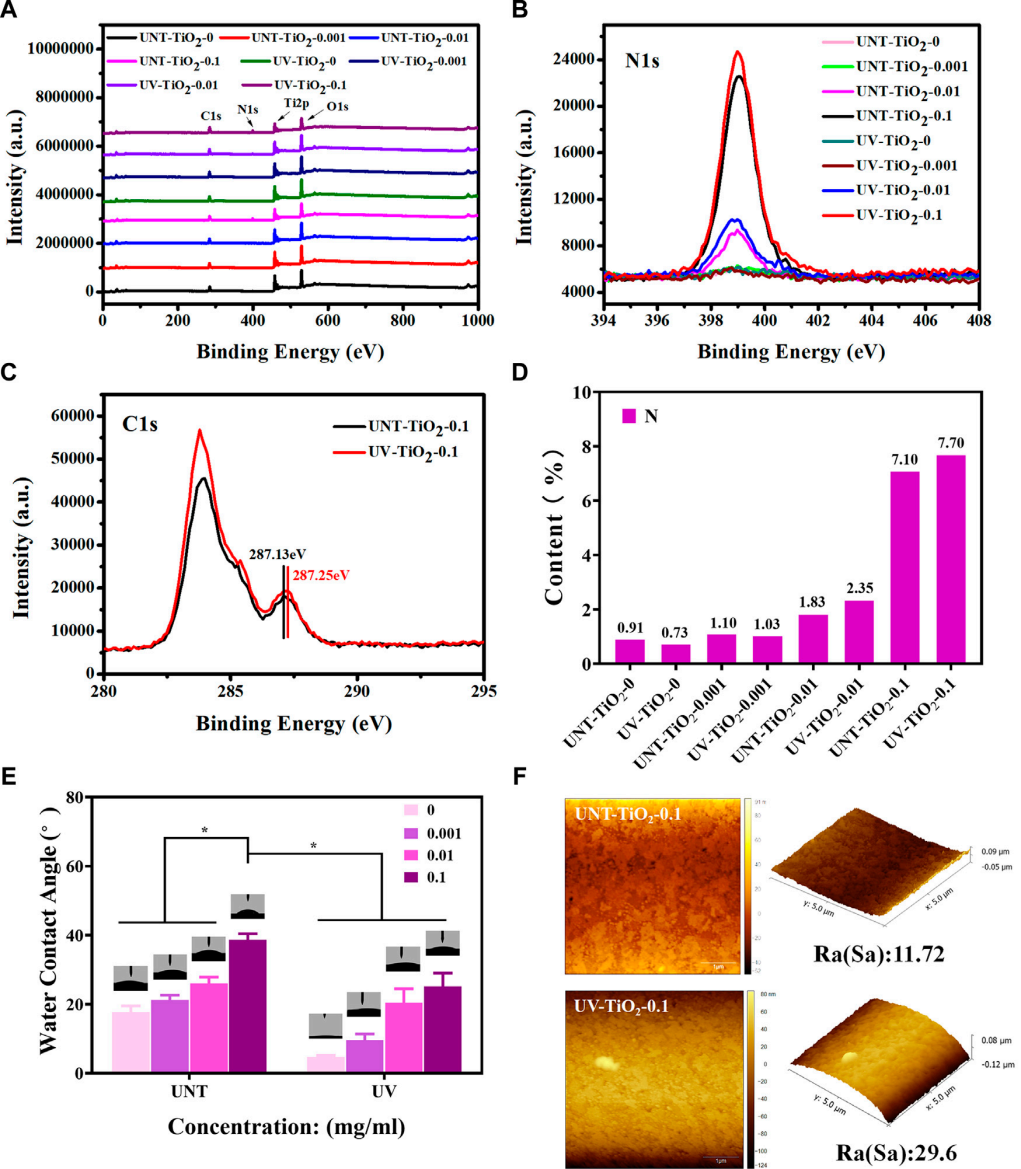
**(A)** XPS full spectrum of TiO_2_ surface modified with EpCAM antibodies. **(B)** XPS high-resolution spectra of N1s on TiO_2_ surface modified with EpCAM antibodies. **(C)** XPS high-resolution spectra of C1s on TiO_2_ surface modified with EpCAM antibodies. **(D)** The statistical plot of the N element content of the TiO_2_ surface modified with EpCAM antibodies. **(E)** The water contact angle of TiO_2_ surface modified with EpCAM antibodies. **(F)** AFM image of TiO_2_ surface modified with EpCAM antibodies. Data were expressed as mean ± standard deviation (n = 3) and analyzed using a one-way ANOVA, *p < 0.05.

As the EpCAM antibodies contain the characteristic element nitrogen (N), XPS was used to detect the atomic percentage of N on the surface of the sample to semi-quantitatively calculate the number of antibodies bound on the sample surface. Figure 3A showed the XPS full spectrum, Figure 3B showed the high-resolution spectra of N1s, and Figure 3D showed the N element content statistics. The results revealed that the N element content of the TiO_2_ surface modified with EpCAM antibodies showed the following order: UV-TiO_2_-0.1 > UNT-TiO_2_-0.1 > UV-TiO_2_-0.01 > UNT-TiO_2_-0.01, indicating that UV-TiO_2_ was able to adsorb more antibodies compared to the UNT-TiO_2_ when immersed in the same concentration of antibody solution. Meanwhile, the N element content of UNT-TiO_2_-0.001 and UV-TiO_2_-0.001 was similar to that of UNT-TiO_2_-0 and UV-TiO_2_-0. This might be due to the adsorbed antibodies in the TiO_2_-0.001 groups being below the XPS device’s detection limit; Figure 3C showed that the peak of UV-TiO_2_-0.1 was higher than the UNT-TiO_2_-0.1, further proving that there were more antibodies adsorbed to UV-TiO_2_-0.1. And they both had a small spike at about 287 eV binding energy, which was attributed to the presence of oxygen-containing hydrocarbons and could be assigned to the -COOH group (Aita et al., 2009; Att et al., 2009). Compared with the small peak of UNT-TiO_2_-0.1 (287.13 eV), the small peak of UV-TiO_2_-0.1 (287.25 eV) was shifted to the right, indicating that the -COOH group lost hydrogen and might be absorbed on the TiO_2_ surface in a bidentate binding structure. UV irradiation can lead to various physicochemical changes in the TiO_2_ surface, including photo-induced superhydrophilicity (Wang et al., 1997; Takeuchi et al., 2005). As shown in Figure 3E, the results showed that the water contact angle of the unirradiated TiO_2_ surface (UNT-TiO_2_-0) was approximately 17.93° ± 1.59°. In comparison, the water contact angle of the UV-irradiated TiO_2_ surface (UV-TiO_2_-0) was approximately 4.9° ± 0.3°, because of the fact that the UV irradiation causes the TiO_2_ surface to become superhydrophilic. The hydrophilic surface is conducive to keeping its activity (Giacomelli et al., 1999). The water contact angles on all the TiO_2_ surfaces increased after the addition of EpCAM antibodies and were positively correlated with the antibody concentration. At the same antibody concentration, the water contact angle of the UV-TiO_2_ groups was lower than that of the UNT-TiO_2_ groups, indicating that the antibodies adhered to the UV-TiO_2_ groups, compared to the UNT-TiO_2_ groups, exposed to fewer hydrophobic terminal.

As shown in Figure 3F, the AFM results showed that the surface roughness of UNT-TiO_2_-0.1 and UV-TiO_2_-0.1 was 11.72 nm and 29.6 nm, respectively. UV-TiO_2_-0.1 had a higher roughness than UNT-TiO_2_-0.1, indicating that the UV-treated TiO_2_ could promote antibodies’ binding to the TiO_2_ surface.

### 3.2 *In vitro* capture of CTCs

**FIGURE 4:**
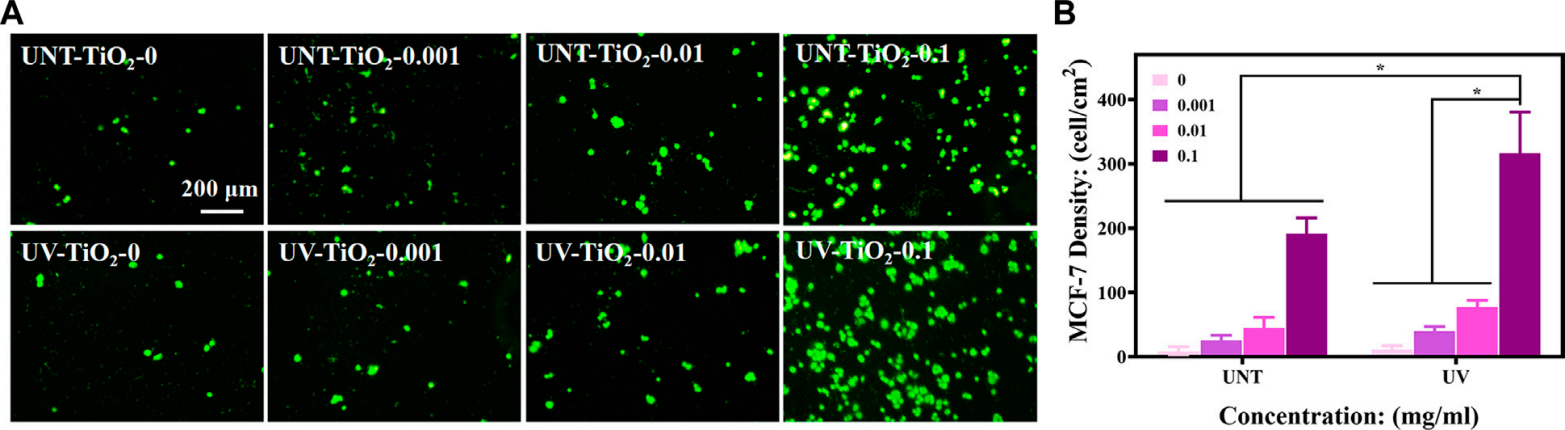
**(A)** Fluorescent images of different sample groups after capturing MCF-7 cells. **(B)** Plots of statistical analysis of the number of MCF-7 cells for **(A)**. Data were expressed as mean ± standard deviation (n = 3) and analyzed using one-way ANOVA, *p < 0.05.

As epithelial cell adhesion molecule (EpCAM) is highly expressed in breast cancer cells (Cimino et al., 2010; Chen et al., 2018), MCF-7 cells were used for capture experiments in the study. As shown in Figures 4A ,B, the number of captured cells increased with the increase of antibody concentration in both the UNT-TiO_2_ and UV-TiO_2_ groups. Among all the samples, UV-TiO_2_-0.1 captured the most cells. This result indicated that as the antibody concentration increased, the more EpCAM antibodies adsorbed on the TiO_2_ surface, the more cells were captured. The cells in UV-TiO_2_-0 groups and UNT-TiO_2_-0 groups were probably caused by the natural settling of the cells and occasional contact. Moreover, at the same concentration, the UV-TiO_2_ groups could capture about 1.5 times more MCF-7 cells than the UNT-TiO_2_ groups. The result indicated that the photo-functionalized TiO_2_ surface modified with EpCAM antibodies could efficiently capture CTCs from the environment *in vitro*.

### 3.3 Dynamic capture of CTCs *in vitro*

To explore the capture efficiency of MCF-7 cells by EpCAM antibodies-modified TiO_2_ in the blood flow state and eliminate the influence of complex components in blood on the capture of MCF-7 cells, this experiment would use the chandler loop system to simulate blood circulation.

**FIGURE 5:**
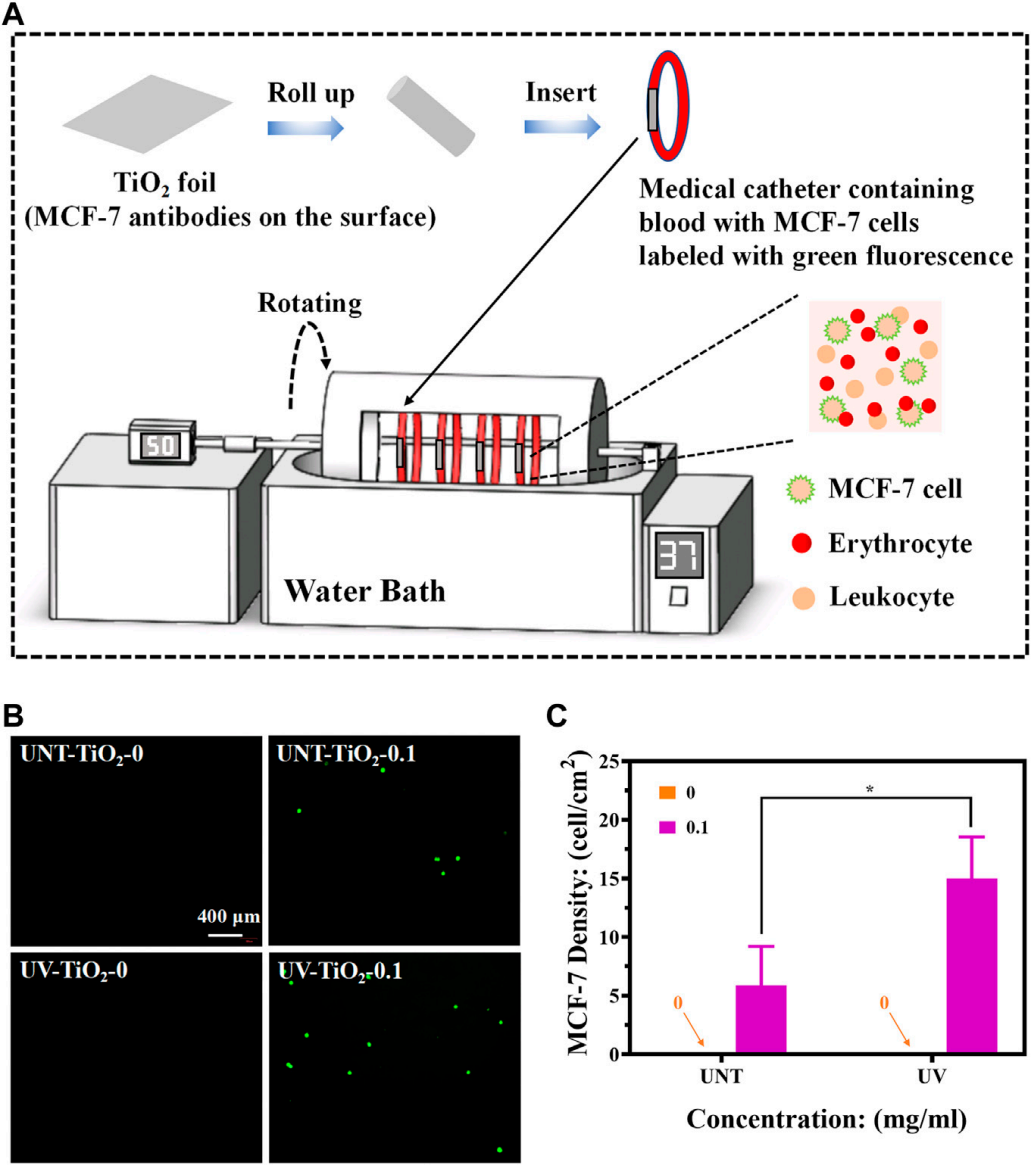
**(A)** Schematic diagram of capturing MCF-7 cells using the chandler loop system device (MCF-7 cells labelled with green fluorescence). **(B)** Fluorescence images of different sample groups after capturing MCF-7 cells. **(C)** Plot of statistical analysis of the number of MCF-7 cells for **(B)**. Data were expressed as mean ± standard deviation (n = 3) and analyzed using one-way ANOVA, *p < 0.05.

As shown in Figures 5B, C, the TiO_2_-0 groups could not capture MCF-7 cells in flowing blood conditions. The EpCAM antibodies-modified TiO_2_, either UV-TiO_2_-0.1 or UNT-TiO_2_-0.1 group, could successfully capture MCF-7 cells. Notably, in the above *in vitro* capture results, the UV-TiO_2_-0.1 groups captured only approximately 165% more MCF-7 cells than the UNT-TiO_2_-0.1 groups, but in the fluid conditions, the UV-TiO_2_-0.1 groups captured approximately 252% more MCF-7 cells than the UNT-TiO_2_-0.1 groups. The reason for the difference could be that some of the antibodies on the unirradiated TiO_2_ surface were easily washed away due to physical adsorption under fluid conditions. In contrast, antibodies adsorbed on the Photo-functionalized TiO_2_ surface had a strong binding force that resisted fluid washout and captured the cells in the fluid. However, the number of MCF-7 cells captured by UV-TiO_2_-0.1 and UNT-TiO_2_-0.1 groups in this experiment was far less than that of MCF-7 cells captured *in vitro* mentioned above. The possible reason was that in the complex whole blood condition, blood cells in the blood obstructed the contact of MCF-7 cells with antibodies on the TiO_2_, resulting in insufficient contact of MCF-7 cells with TiO_2_. In conclusion, the above results demonstrated that photo-functionalized TiO_2_ surfaces modified with EpCAM antibodies could efficiently capture CTCs from the environment *in vitro*.

### 3.4 *In vivo* capture of CTCs

Furthermore, we constructed a rabbit model to simulate human blood circulation to determine whether TiO_2_ modified with EpCAM antibodies could capture MCF-7 cells *in vivo*.

**FIGURE 6:**
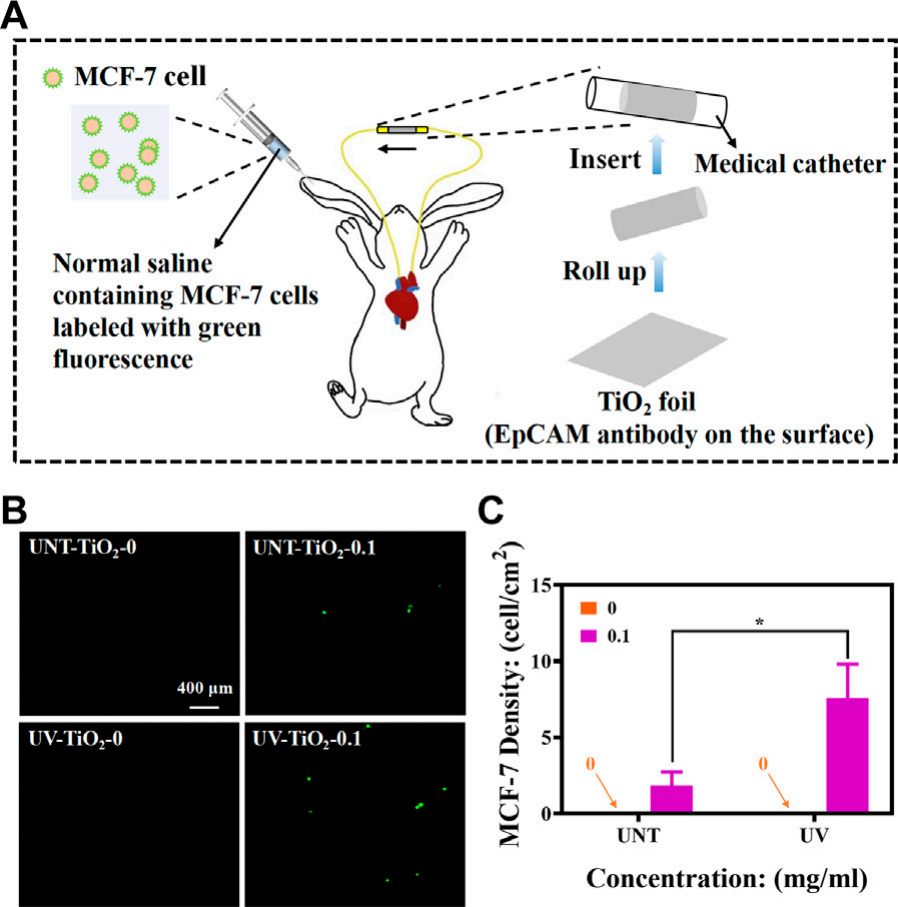
**(A)** Schematic diagram of *in vivo* MCF-7 cell capture using New Zealand white rabbits (MCF-7 cells labelled with green fluorescence). **(B)** Fluorescence images of different sample groups after capturing MCF-7 cells. **(C)** Plot of statistical analysis of the number of MCF-7 cells for **(B)**. Data were expressed as mean ± standard deviation (n = 3) and analyzed using one-way ANOVA, *p < 0.05.

As shown in Figure 6A, a medical catheter containing TiO_2_ modified with EpCAM antibodies was used to connect the rabbit’s carotid artery and jugular vein to construct a closed circulatory system. MCF-7 cells labelled with CFDA SE stain (emitting green fluorescence) were then injected into the body from the rabbit’s ear vein, and the MCF-7 cells were captured as the blood flowed through the TiO_2_ modified with EpCAM antibodies. As shown in Figures 6B, C, the TiO_2_-0 groups could not capture MCF-7 cells *in vivo*. In contrast, after the immobilization of EpCAM antibodies, TiO_2_ could effectively capture MCF-7 cells, and UV-TiO_2_-0.1 captured about 4 times more MCF-7 cells than UNT-TiO_2_-0.1. However, the number of cells captured by both was less than that of cells captured *in vitro*, probably because the rabbit’s immune system rejected the foreign bodies and cleared some MCF-7 cells. These results indicated that photo-functionalized TiO_2_ with EpCAM antibodies could capture CTCs from the dynamic environment *in vivo*.

## 4 Conclusion

In summary, we have constructed a new platform that significantly increased the capture efficiency of CTCs by bonding EpCAM antibodies with electrostatic mechanisms based on the charge change on the TiO_2_ surface caused by UV irradiation, which exposed more binding sites for antibodies bound to the TiO_2_ surface. Our experimental results also showed that the photo-functionalized TiO_2_ modified with EpCAM antibodies could efficiently capture CTCs from environments *in vitro* and *in vivo*. Since TiO_2_ can be deposited on the surface of various inorganic materials by physical vapor deposition and has excellent biocompatibility. Therefore, the method may be suitable for the construction of a variety of various materials for the capture of CTCs *in vitro* (such as magnetic beads and silicon-based photoelectrochemical platforms) and *in vivo* (stainless steel indwelling needles).

## Data Availability

The original contributions presented in the study are included in the article/Supplementary Material, further inquiries can be directed to the corresponding authors.
